# The Poetic Wavelength—Tuning into the Meaningful Poetics of Psychosis

**DOI:** 10.1007/s10912-024-09896-4

**Published:** 2024-09-12

**Authors:** Mark Pearson

**Affiliations:** School of Health Sciences, Queen’s Medical Centre, The University of Nottingham, Nottingham, NG7 2HA UK

**Keywords:** Poetry, Creative writing, Psychosis, Narratives

## Abstract

Despite the emerging evidence base to support the therapeutic potential of creative writing and poetry for a variety of mental health problems, the therapeutic potential of poetry for people who have experienced psychosis remains poorly understood. The paper argues that by considering psychosis as meaningful poetics, this epistemological shift has the potential to foster curious inquiry and increase opportunities for meaningful dialogue. The paper introduces and explores the concept of the ‘poetic wavelength’, building on the previously established notion of the psychotic wavelength, which proposes that others need to ‘tune in’ to what is being communicated through psychosis. The concept of the poetic wavelength suggests that the reading and writing of poetry may support this process of ‘tuning in’ both for those experiencing psychosis and those working therapeutically with them.

## Introduction

Despite the significant evidence base to support the psychotherapeutic value of poetry and creative writing for people experiencing a range of mental health problems (Frattaroli 2006; Mazza 2017; Pennebaker & Beall 1986; Pennebaker & Smyth 2016), including conditions described as severe mental illness (Chiang et al., 2019; King et al., 2013), there remains a deficit of research, and potential under appreciation, of the therapeutic value of poetry for people who have experienced psychosis. This is despite poets having long been associated with psychosis (Holm-Hadulla et al., 2010; Mason et al., 2015). Sigmund Freud, who arguably wrote very poetically (Curtis 2013), was significantly influenced by the poets such as Johann Wolfgang Von Goethe and proposed that poets and psychoanalysts are drawn to similar existential examinations of life (Kristiansen 2013).

Poetry and creative writing share some commonalities with other creative participatory arts and are often utilised in collaboration with visual methods such as photography or drawing (Miller & Zelenko 2022). These creative practices not only document ideas or moments but also advocate for a creative approach to capturing experiences, wherein ideas can be ‘shown and felt, not merely told’ (Batty & Baker 2018, 2). The reading and writing of poetry provides an opportunity to craft narratives based on one’s thoughts, feelings, and attitudes (Kuriakose & Jena, 2024; Pearson et al., 2024). It is through these stories that meanings can be explored, discovered, and developed. Han (2024) writes that ‘by placing our sorrow under a narrative light…a story raises them above mere facticity. Instead of solidifying as a mental block, they liquefy in the narrative flow’.

The concept of the poetic wavelength is underpinned by research exploring the relationship between poetry and psychosis and developed as part of a PhD thesis (Pearson et al., 2020, 2022; Pearson 2023). This research involved the development of a conceptual framework exploring the relationship between poetry and psychosis and the collection of qualitative data through narrative interviews with two groups of people: firstly, people with lived experience of psychosis who read and/or write poetry and secondly practitioners using poetry in their therapeutic work with people who have experienced psychosis. The results suggested that the reading and writing of poetry may help people who are experiencing psychosis to narrate their experiences and may support those working within mental health services to more effectively understand and engage with what is being communicated. This paper seeks to explore the notion of psychosis as meaningful poetics and subsequently introduce the notion of the poetic wavelength.

## Psychosis as meaningful poetics

Emil Kraepelin and Eugen Bleuler are described as arguably the most influential individuals in relation to the traditional classification of schizophrenia (Read 2013). Their work developed the original nosological classification of psychosis and laid the foundations for a biomedical orthodoxy which positioned the utterances of people with psychosis as illogical, incoherent, and merely representative of an underlying thought disorder. Whilst mental health practice has obviously developed significantly since this initial conceptualisation of psychosis, there is a legacy which remains from these historical interpretations, observable in the way contemporary mental health professionals can remain reluctant or uncertain when engaging in dialogue with individuals about their experiences which are described as psychosis (Farrelly & Lester 2014; McCabe et al., 2002; McCabe & Priebe 2008).

Psychosis or madness is often represented as something which needs to be supressed and managed by reason and logical thought (Kusters 2020). This management has traditionally involved the pathologisation of language, wherein the perceived irrationality of the individual’s utterances become conceptualised as evidence of some form of mental illness. Kusters (2020) refers to the verbal impotence experienced by people described as psychotic, as in their attempts to narrate their experiences using figurative and creative language, their utterances are perceived and labelled as incoherent. However, it is a false assumption that language is a homogenous entity, with stable omnipresent rules of coherence (Thomas 1997), in which sanity or madness can be defined linguistically. Interestingly, examples of what might be considered linguistic abnormalities such as ‘clanging’, meaning combining words in a sentence based on sound rather than meaning, or the creation of ‘neologisms’ or new words are frequently utilised in the assessment and diagnoses of psychosis (Covington et al., 2005). These linguistic devices are considered diagnostic indicators within mental health services, and yet both of these are frequently observed and considered creatively legitimate within poetry, and especially in relation to neologisms, outside of a psychiatric setting are often appreciated as meaningful expressions bound up in social and political contexts (ten Hacken & Koliopoulou, 2020).

In using the term meaningful poetics, the aim is not to glamorise psychotic experiences, or to fail to appreciate the potential suffering of those who experienced psychosis (Pearson 2023). Rather, the aim is to propose a conceptualisation of psychosis which relates to the way in which Campbell (2005, 63) describes poetics as ‘specialised languages that are embedded in, but distinct from, natural language, that draw attention to their own differences from natural language, and that work within larger systems of meaning…’. This seems rather apt in relation to psychosis, as both poetry and psychosis are often manifest in language (Patterson 2014), sometimes marked by communication that does sit within larger linguistic landscapes, but which is unique and largely defined by the way in which it calls attention to a departure from what might be considered common sense (Campbell 2005; Pearson, 2023).

Wilkinson (2009) discusses the way in which ‘poetic’ is often an axiom for some indescribable process, communicating something which otherwise would have remained incommunicable. Both poetry and psychosis are orientated by what cannot be said, or what remains unsayable, with meanings often indirectly expressed (Franke 2014; Wilkinson 2009). In the context of psychosis, this struggle with the incommunicable may represent an embodied process of trying to narrate a previous trauma and an altered sense of self or identity in response to lived experiences (Bentall et al., 2012; Corsten & Longden 2013). Romm et al. (2023) observed that following a twelve-week course in creative writing, adults with psychosis were able to develop a sense of connectedness, mastery, and empowerment. Therefore, the crafting of these narratives can be considered a poetic process, wherein the author is no longer a passive victim afflicted by a disorder, but rather an individual engaged in an ongoing meaning making process (Leonhardt et al., 2015).

Language is the primary system through which individuals narrate and make meaning of their experiences (Anderson & Goolishian 1988). The narratives which are crafted in response to psychosis are one of the primary tools available to people in attempting to reclaim a sense of agency within their lives (Davidson & Strauss 1992) and are an essential component in the process of recovery (Roe & Davidson 2005). Through the construction of narratives, new understandings can emerge in relation to how the current experiences may be linked to past traumas or distress (Seikkula 2022). The importance of narratives is long established within poetry; Seamus Heaney (2000, 7) suggested that the writing of poetry ‘involves not only a poet’s way with words, his management of metre rhythm and verbal texture; it involves also a definition of his stance towards life, a definition of his own reality’. This defining of one’s own reality and identity has the potential to be especially salient for people who have experienced psychosis, especially if elements of that reality have been diminished, silenced, or ignored.

## The poetic wavelength

Lucas (1993) proposed that rather than being incoherent, psychotic communication exists on what he calls the ‘psychotic wavelength’, and it is therefore the role of those working with people experiencing psychosis, to ‘tune in’ to this wavelength in order to understand what is being communicated. The analogy of an FM radio illustrates this process of tuning in, considering the way in which attempting to find a radio signal often involves firstly only hearing the crackle of white noise, but eventually the sounds start to cohere with voices or music emerging from what was previously experienced as meaningless noise. The concept of the poetic wavelength seeks to develop this theory of ‘tuning in’, proposing that the reading and writing of poetry may help to support this process of tuning in to the meaningful poetics of psychosis (Pearson et al., 2020, 2022).

This poetic way of working requires a repositioning toward a hermeneutic phenomenological exploration and understanding of psychosis, appreciating that ‘moods and patterns of behavior do not emerge exclusively from dysfunctions in the brain but from the ways in which the self is meaningfully engaged in the world’ (Aho, 2008, 254). Psychosis can be a severely distressing personal crisis involving intense feelings of shame, fear, and confusion (Bögle & Boden, 2022). However, as suggested by Wharne (2015), although psychosis can create many problems for an individual, one of the most difficult is negotiating a shared understanding with others.

It is the function of both poetry and therapy to preserve a space in which people can tell and share their stories (McLeod 1997) and to provide the opportunity to give words to the unsayable. Thus, it is essential that these storied poetic utterances are responded to dialogically, echoing the assertion by Read (2019) that the most effective approaches to working therapeutically with people experiencing psychosis is based on a humane understanding that validates the individual’s psychosis as meaningful and inextricably linked to their past experiences. Seikkula (2022, 58) emphasises that all psychotic experiences should be respected without conditions, in order to ‘deepen the speaker’s awareness and understanding of what they are saying, by taking it seriously’. This may be a contrast to ‘reality orientated’ approaches (Pratiwi & Dewi 2016), which may seek to challenge an individual to evaluate the reality of their experiences. Whilst this attempt at reorientation may be helpful in some situations, attempting to ground experiences of psychosis within an objective reality may be unhelpful for the person, cause further distress, and create further distance between them and the therapist (Avdi 2005).

Evans (2008, 98) proposes that the priority for any mental health practitioner working with someone experiencing psychosis should be to develop ‘dialogue with meaning’. The concept of the poetic wavelength has the potential to increase opportunities for dialogue by orientating practice towards a position which is primarily curious and dialogical (Pearson 2023). This is potentially antithetical to other processes or paradigms within mental health services which can reduce curiosity as a by-product of imposing larger meta-narratives upon individual narratives (Evans 2008). This diminishment of curiosity is a potentially pernicious process which results in traits becoming conceptualised as rigid and constant within the identity of individuals (Pearson 2023). For the individual, this imposition of meta-narratives might be experienced as an immersion within the potentially pathologising discourses of mental healthcare (Pack 2008), resulting in feelings of inadequacy or hyper self-criticism (Coaston 2020).

In attempting to tune into the poetic wavelength, psychotic experiences can be conceptualised as not a stable element of an individual’s identity but rather a momentary experience which is shaped socially in relation with, and through dialogue with others (White 2004). This is a relational process, and poetry and psychosis can both be considered forms of relational expressions, reflecting the way a person experiences themselves in relation to the world around them in a way which is rich in metaphor and symbolism (Wilkinson 2009).

This relationship between the abstract and the concrete enables people to discuss traumatic memories or thoughts indirectly (Kopp 2013), as can be observed in the way that people are more likely to use metaphors when discussing intense emotional experiences (Fainsilber & Ortony 1987). Metaphors ‘reflect and reinforce particular ways of making sense of subjective and sensitive experiences’ (Demjén et al., 2019, 17) and thus have a significant tradition within psychotherapeutic practice (Kopp 2013; Turner 2014). In speaking of ‘the metaphorical process’, Fiumara (2005, 12) proposes that metaphors ‘invite, direct and control exploration…in which new knowledge is implicit, though not yet manifest’. For those experiencing psychosis, much of the knowledge about their experience may still be manifesting; however, metaphors can serve as a bridge between the inner subjective experience and the external understanding and comprehension of interlocutors (Mould et al., 2010).

The exploration of psychosis can be highly distressing, and this remains true even in the context of a more poetic understanding. Pennebaker and Smyth (2016) comment that when writing about emotive topics, individuals may initially experience significant distress before they start to experience a sense of catharsis. However, whilst poetry, or any form of artistic expression, has the potential to overwhelm authors (Levine & Levine 1999), the crafting of a poem may help to create a sense of psychological distance between an individual and the pain they are experiencing. Lippi et al. (2016, 775) propose that ‘writing can be understood as a means of separating oneself from the invasive presence of the persecutory other…’. Thus the poem itself becomes a container for intense pain, it is ‘a holding place for experiences which are otherwise hard to hold’ (Davis & Billington 2016, 406). This function of containment potentially offered by a poem is analogous with the function of the psychotherapist and their provision of containment to an individual, a process through which the therapist or therapeutic relationship becomes the container for what is experienced as intolerable by an individual (Szykierski 2010; Winship & MacDonald 2018; Pearson 2023).

## Conclusion

This paper has proposed the notion of the poetic wavelength and how this may assist those who work within mental health services to consider the meaningful poetics of psychosis. One of the most fundamental properties of poetry is to provide a way of exploring what it is to be human, to consider the way identity is shaped, maintained, and transformed through our narratives (Faulkner 2009; Jeffs and Pepper, 2005; Pearson et al., 2020). However, so many of the narratives of those who experience psychosis remain ignored or underappreciated, with some of those working within contemporary mental health services remaining reluctant or uncertain as to how to engage in dialogue with service users about their psychotic symptoms (Farrelly & Lester, 2014; McCabe et al., 2002; McCabe & Priebe 2008).

In the case of psychosis, which may be a response to overwhelming trauma (Seikkula 2022; Van Werde 2021), there may well be a fear of giving words to such experiences as the process of being re-presented with the content of the narrative can be re-traumatising (Cole 2010). Whilst poetry cannot take away this fear, it can offer a space for individuals to explore and share their narratives, in an authentic and deeply human way, and offer the opportunity for people to craft new stories which are rich in meaning.
